# Failure to rescue patients after emergency laparotomy for large bowel perforation: analysis of the National Emergency Laparotomy Audit (NELA)

**DOI:** 10.1093/bjsopen/zraa060

**Published:** 2021-02-20

**Authors:** O Peacock, F Yanni, A Kuryba, D Cromwell, S Lockwood, I Anderson, R S Vohra, NELA Project Team, P Cripps, N Eugene, J Goodwin, S Hare, M Grocott, E Davies, M Bassett, C Johnston, J Lourtie, R Moonesinghe, D Murray, D Papadimitriou, C Peden, T Poulton, T Salih, K Walker, S Warren, M Cripps, S Drake, M Oliver

**Affiliations:** Peter MacCallum Cancer Centre, Melbourne, Victoria, Australia; Trent Oesophago-Gastric Unit, Nottingham City Hospital Campus, Nottingham University Hospitals NHS Trust, Nottingham, UK; Clinical Effectiveness Unit, Royal College of Surgeons of England, London, UK; Clinical Effectiveness Unit, Royal College of Surgeons of England, London, UK; Department of Health Services Research and Policy, London School of Hygiene and Tropical Medicine, London, UK; Colorectal Surgery Department, Bradford Royal Infirmary, Bradford Teaching Hospitals NHS Foundation Trust, Bradford, UK; University of Manchester School of Medicine, Salford Royal NHS Foundation Trust, Salford, UK; Trent Oesophago-Gastric Unit, Nottingham City Hospital Campus, Nottingham University Hospitals NHS Trust, Nottingham, UK

## Abstract

**Background:**

Past studies have highlighted variation in in-hospital mortality rates among hospitals performing emergency laparotomy for large bowel perforation. The aim of this study was to investigate whether failure to rescue (FTR) contributes to this variability.

**Methods:**

Patients aged 18 years or over requiring surgery for large bowel perforation between 2013 and 2016 were extracted from the National Emergency Laparotomy Audit (NELA) database. Information on complications were identified using linked Hospital Episode Statistics data and in-hospital deaths from the Office for National Statistics. The FTR rate was defined as the proportion of patients dying in hospital with a recorded complication, and was examined in hospitals grouped as having low, medium or high overall postoperative mortality.

**Results:**

Overall, 6413 patients were included with 1029 (16.0 per cent) in-hospital deaths. Some 3533 patients (55.1 per cent) had at least one complication: 1023 surgical (16.0 per cent) and 3332 medical (52.0 per cent) complications. There were 22 in-hospital deaths following a surgical complication alone, 685 deaths following a medical complication alone, 150 deaths following both a surgical and medical complication, and 172 deaths with no recorded complication. The risk of in-hospital death was high among patients who suffered either type of complication (857 deaths in 3533 patients; FTR rate 24.3 per cent): 172 deaths followed a surgical complication (FTR-surgical rate 16.8 per cent) and 835 deaths followed a medical complication (FTR-medical rate of 25.1 per cent). After adjustment for patient characteristics and hospital factors, hospitals grouped as having low, medium or high overall postoperative mortality did not have different FTR rates (*P* = 0.770).

**Conclusion:**

Among patients having emergency laparotomy for large bowel perforation, efforts to reduce the risk of in-hospital death should focus on reducing avoidable complications. There was no evidence of variation in FTR rates across National Health Service hospitals in England.

## Teaser

The aims of this study were to evaluate rates of complications, failure to rescue (FTR) and interprovider differences in patients undergoing emergency laparotomy for large bowel perforation in England. The FTR-surgical rate was 16.8 per cent and the FTR-medical rate was 25.1 per cent. FTR rates following emergency laparotomy for large bowel perforation are high, with no evidence of variation between hospitals.

## Resumen

**Antecedentes:**

Estudios anteriores han puesto de manifiesto la variación en las tasas de mortalidad hospitalaria entre los hospitales que realizan laparotomía de urgencia por perforación de colon. El objetivo de este estudio fue investigar si el fracaso en el rescate (*failure to rescue*, FTR) contribuye a esta variabilidad.

**Métodos:**

Los pacientes de 18 años o más que requirieron cirugía por perforación del intestino grueso entre 2013 y 2016 se extrajeron de la base de datos de la *National Emergency Laparotomy Audit* (NELA). La información sobre las complicaciones se identificó utilizando datos vinculados de estadísticas de episodios hospitalarios (*Hospital Episode Statistics*, HES) y muertes intrahospitalarias de la Oficina de Estadísticas Nacionales (*Office for National Statistics*, ONS). La tasa de FTR se definió como la proporción de pacientes que fallecieron en el hospital con una complicación registrada, analizándose en los hospitales agrupados según la mortalidad postoperatoria general fuera baja, media o alta.

**Resultados:**

En total, se incluyeron 6.413 pacientes con 1.029 (16,0%) muertes intrahospitalarias. Un total de 3.533 (55,1%) pacientes tuvieron al menos una complicación: 1.023 (16,0%) una complicación quirúrgica y 3.332 (52,0%) una complicación médica. Hubo 22 muertes intrahospitalarias después de solo una complicación quirúrgica, 685 muertes después de solo una complicación médica, 150 muertes después de una complicación quirúrgica y médica y 172 muertes sin ninguna complicación registrada. El riesgo de mortalidad hospitalaria fue alto entre los pacientes que sufrieron cualquier tipo de complicación (857 muertes; tasa de FTR 24,3%): 172 muertes siguieron a una complicación quirúrgica (tasa de FTR-quirúrgica 16,8%) y 835 muertes siguieron a una complicación médica (tasa FTR-médica del 25,1%). Tras el ajuste de las características de los pacientes y los factores hospitalarios, los hospitales agrupados en mortalidad postoperatoria global baja, media o alta no tuvieron tasas de FTR diferentes (*P* = 0,770).

**Conclusión:**

Entre los pacientes que se someten a laparotomía de urgencia por perforación del intestino grueso, los esfuerzos para reducir el riesgo de muerte intrahospitalaria deben centrarse en reducir las complicaciones evitables. No hubo evidencia de variación en las tasas de FTR entre los hospitales del NHS en Inglaterra.

## Introduction

In England and Wales annually, almost 30 000 patients undergo an emergency laparotomy, 20 per cent for bowel perforation^1^. However, this procedure is associated with a postoperative mortality rate of up to 33 per cent^2–7^, possibly due to the prevalence of complications following laparotomy. There is growing evidence that postoperative complication and reintervention rates should form a key quality metric^8^^,^^9^, and the prevention, early detection and timely instigation of appropriate management of complications might more closely reflect processes of care within healthcare organizations, rather than postoperative mortality alone^10^^,^^11^. This has developed into the concept of failure to rescue (FTR), which is the proportion of patients who die after suffering a complication^12^.

The National Emergency Laparotomy Audit (NELA) is an ongoing national clinical audit designed to evaluate the processes of care and outcomes of patients undergoing emergency bowel surgery in National Health Service (NHS) hospitals in England and Wales^1^. It aims to provide comparative information for hospital benchmarking, and thereby support local quality improvement initiatives to improve the quality of care and outcomes for patients undergoing emergency bowel surgery. The data set contains comprehensive preoperative, perioperative and postoperative demographic and outcome data of more than 100 000 patients who have undergone emergency laparotomy since December 2013. Despite several studies investigating FTR following elective abdominal surgery, there are currently few data evaluating the FTR metric in emergency surgery^13^. The present study used data from NELA for a secondary analysis to: describe rates of in-hospital mortality in patients undergoing emergency laparotomy for large bowel perforations in England; evaluate postoperative complications and FTR rates; and investigate whether FTR rates vary between hospitals.

## Methods

The study used an extract from the NELA data set that was linked to the Hospital Episode Statistics (HES) database^14^ and the Office for National Statistics (ONS) death register. Data are submitted by NHS hospitals in England; the process is described in detail in the NELA annual report^1^. NELA is approved under section 251 of the NHS Act 2006 by the Confidential Advisory Group. The study was approved by Healthcare Quality Improvement Partnership.

The HES database^14^, which contains details of all admissions to NHS hospitals in England, was used to obtain information on patient co-morbidity, postoperative complications, and procedures that occurred in the period following the initial emergency laparotomy. Systematic reviews have shown that primary diagnostic and procedural coding accuracy in HES is sufficiently robust for the purpose of observational research^15^ and that specific patient cohorts can be identified with a high degree of accuracy^16^.

*Study population*

Patients aged 18 years or over requiring a primary emergency laparotomy for large bowel perforation in an NHS hospital in England between 2013 and 2016 were included. Patients admitted to hospitals in Wales were excluded, owing to the data not being linked with HES.

Patients were eligible if the recorded indication for surgery was perforation and/or peritonitis, with the primary procedure recorded as a large bowel resection (colectomy or Hartmann procedure), drainage of abscess or collection, or stoma formation. All causes of large bowel perforation, such as diverticulitis, malignancy and colitis, were included.

Patients requiring an emergency laparotomy during an elective admission or recorded as undergoing a surgical procedure within the previous 30 days were excluded, as their management was unlikely to be representative of patients requiring an emergency laparotomy as the index procedure within an emergency admission. Patients who did not have an emergency laparotomy recorded in HES on the date of operation recorded in NELA were also excluded,

### Outcome definitions

The primary outcome measures were in-hospital mortality and FTR rates for all patients undergoing an index emergency laparotomy for large bowel perforations at all NHS hospitals in England. Information on the date of death was based on ONS date of death or, if this was not available, the HES discharge status. Complications were defined and selected with reference to OPCS-4 codes used in previously published FTR studies^17^^,^^18^. Surgical complications were defined by reoperations or reinterventions occurring in the same HES hospital admission as the index NELA procedure, but on a subsequent date. Interventions were further classified by the type of procedure; upper digestive tract (OPCS-4, code G), lower digestive tract (code H), other abdominal organs (principally digestive; code J), skin (code S) or soft tissue (code T), and interventional radiological procedures (code T45) (*Table S1*). Medical complications were defined by ICD-10 diagnosis codes, using a method described previously^17^ (*Table S1*). Complications were treated as binary outcomes (present or absent).

The FTR rate was defined as the number of patients dying in hospital with a recorded complication divided by the total number of patients with a recorded complication. The uncomplicated mortality rate was the number of patients dying without recorded complications divided by the total number of patients without a recorded complication. FTR was considered in three parts: the overall FTR rate; a measure of surgical complications (FTR-S); and a measure of medical complications (FTR-M).

### Patient characteristics

The following patient-level variables were obtained from the NELA data set: demographic (age on admission, sex) and preoperative (urgency of surgery, ECG findings, number of operations within the admission, previous cardiac and respiratory history, ASA fitness grade, Portsmouth (P) POSSUM-predicted mortality^19^, physiological (serum sodium, potassium, urea, creatinine, haemoglobin, white cell count (WCC), lactate, pulse, systolic BP, and Glasgow Coma Scale (GCS) score) and perioperative (operative severity, procedure performed, operative approach, intraoperative findings, level of contamination, blood loss and postoperative destination). Service provision and hospital variables (day of the week that surgery was performed, volume of cases performed each year, unplanned critical care admission, specialty of surgeon performing initial operation or grade of surgeon, consultant level present or not, consultant review within 14 h) were also extracted from the NELA data set. Information on co-morbidity was summarized with the Royal College of Surgeons Charlson Score^20^, which used information on conditions coded within HES records.

### Statistical analysis

Descriptive results of patient variables are presented as mean(s.d.), median (i.q.r.) or number (percentage) values as appropriate. The χ^2^ test was used to test the differences between categorical demographic variables. A logistic regression model was used to analyse the relationship between postoperative mortality and medical/surgical complication rates, after adjusting for patient and hospital risk factors. A multiple variable survival model was used to remove the effect of potential confounders.

Adjusted in-hospital level mortality rates for each NHS hospital were calculated using indirect standardization, with the NELA risk adjustment model^21^ being used to produce the predicted number of deaths. The model included the following variables: age, sex, audit year of procedure, urgency of the surgery, ECG findings, number of operations within the admission, cardiac signs, respiratory history, ASA fitness grade, serum sodium, potassium, urea, creatinine, haemoglobin, WCC, pulse, systolic BP, GCS, operative severity, peritoneal soiling, intraoperative blood loss, presence of malignancy, as well as interactions between age and ASA grade, and age and respiratory history.

To examine whether FTR rates varied across NHS hospitals, the hospitals were grouped into low-, medium- or high-mortality categories according to their adjusted mortality when compared against the mean mortality. The low-mortality category contained hospitals with a mortality rate equal to or below 1 standard deviation from the mean; the high-mortality category contained hospitals with a mortality rateequal to or above 1 standard deviation from the mean; the remaining hospitals were classified as having medium mortality.

Data were analysed using Stata^®^ SE v15 statistical software (StataCorp, College Station, TX, USA).

## Results

**Fig. 1:**
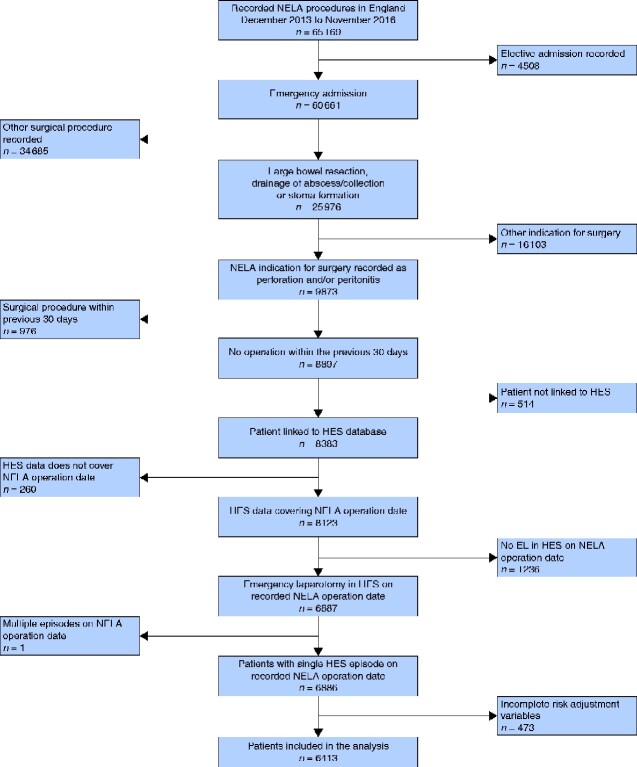
Flow diagram showing the distribution of patient characteristics and operative findings by complication groups NELA, National Emergency Laparotomy Audit; HES, Hospital Episode Statistics; EL, emergency laparotomy.

The NELA database contained information for 65 169 patients who had an emergency laparotomy for bowel surgery in NHS hospitals in England and Wales between 1 December 2013 and 30 November 2016. During this period, 6886 patients (10.6 per cent) underwent emergency laparotomy for a large bowel perforation that could be linked to HES. Of these, 6413 (93.1 per cent) had complete data for risk adjustment and were considered eligible for final analysis (*Fig. 1*).

The baseline characteristics are outlined in *Table 1*. The majority of patients were aged 65 years or over (3644 patients, 56.8 per cent) and there was a slightly higher proportion of women (3485, 54.3 per cent). The majority of patients had preoperative CT (5667, 88.4 per cent) and an open operation (5602, 87.4 per cent), which was performed by a consultant (5810, 90.6 per cent), distributed equally across the days of the week. The median time from admission to consultant review was 10.6 (i.q.r. 4.5–30) h.

**Table 1:** In-hospital mortality by patient characteristics and operative findings

		No. of patients	In-hospital mortality (%)	*P* *
Overall		6413	16.0	
Sex	M	2928	14.7	0.007
	F	3485	17.2	
Age (years)	18–44	794	2.9	<0.001
	45–64	1,975	8.9	
	65–80	2470	19.2	
	> 80	1174	30.3	
Surgical procedure	Colonic resection	5777	15.9	0.240
	Stoma formation	274	19.7	
	Drainage	362	16.0	
Type of colonic resection	Right colectomy	1511	14.9	<0.001
	Left colectomy	452	15.3	
	Hartmann procedure	3168	14.7	
	Subtotal colectomy	646	24.6	
No. of co-morbidities (includes malignancy)	0	2598	8.0	<0.001
	1	1877	17.2	
	2	1267	23.6	
	≥ 3	671	30.0	
ASA grade	I (no systemic disease)	649	1.7	<0.001
	II (mild systemic disease)	1974	4.3	
	III (severe systemic disease, not life-threatening)	2173	13.1	
	IV (severe, life-threatening)	1471	37.3	
	V (moribund)	146	67.8	
Preoperative POSSUM score	< 10	2638	2.3	<0.001
	10–24.9	1526	12.3	
	25–49.9	1106	25.0	
	≥ 50	1143	44.1	
Surgical urgency (h)	> 18	516	7.0	<0.001
	6–18	1533	8.2	
	< 6	4357	19.9	
	Missing	7	0.0	
Indication for surgery	Perforation only	2454	13.9	<0.001
	Peritonitis only	1432	14.5	
	Perforation and peritonitis	2527	19.0	
Actual peritoneal soiling	None	514	11.1	<0.001
	Serous fluid	645	14.3	
	Localized pus	1661	7.5	
	Free pus, blood or bowel contents	3572	21.1	
	Missing	21	9.5	
Total blood loss (ml)	≤500	5797	15.3	<0.001
	> 500	562	24.6	
Postoperative destination	Ward	1450	2.8	<0.001
	HDU	2140	6.4	
	ICU	2810	29.9	

HDU, high-dependency unit.

*χ^2^ test.

The overall in-hospital mortality rate for emergency laparotomy for large bowel perforation was 16.0 per cent (1029 patients). The risk of death was higher among older patients (30.3 per cent in-hospital mortality rate in patients aged over 80 years *versus* 12.9 per cent for those aged 80 years or less; *P* < 0.001), in patients with more co-morbidities (25.8 per cent in-hospital mortality in patients with 2 or more co-morbidities *versus* 11.9 per cent in those with 1 or none; *P* < 0.001), and in patients who had higher ASA and P-POSSUM ratings (*Table 1*).

The overall complication rate was 55.1 per cent, with 3533 patients suffering one or more complications. The medical complication rate (3332 patients, 52.0 per cent) was higher than the surgical complication rate (1023 patients, 16.0 per cent). The likelihood of complications increased with age, ASA grade, number of co-morbidities, P-POSSUM score, urgency of surgery, level of contamination, blood loss and postoperative destination (*Table 2*).

**Table 2:** Distribution of patient characteristics and operative findings by complication group

		Recorded complications group			
		None	Medical only	Surgical only	Both
Overall		2880 (44.9)	2510 (39.1)	201 (3.1)	822 (12.8)
Sex	M	1355 (47.0)	1092 (43.5)	95 (47.3)	386 (47.0)
	F	1525 (53.0)	1418 (56.5)	106 (52.7)	436 (53.0)
Age (years)	18–44	512 (17.8)	172 (6.9)	33 (16.4)	77 (9.4)
	45–64	1009 (35.0)	614 (24.5)	69 (34.3)	283 (34.4)
	65–80	966 (33.5)	1094 (43.6)	74 (36.8)	336 (40.9)
	> 80	393 (13.6)	630 (25.1)	25 (12.4)	126 (15.3)
Procedure	Colonic resection	2605 (90.5)	2267 (90.3)	163 (81.1)	742 (90.3)
	Stoma formation	122 (4.2)	94 (3.7)	19 (9.5)	39 (4.7)
	Drainage	153 (5.3)	149 (5.9)	19 (9.5)	41 (5.0)
Type of colonic resection (*n* = 5777)	Right colectomy (*n* = 1511)	802 (53.1)	493 (32.6)	43 (2.8)	173 (11.4)
	Left colectomy (*n* = 452)	213 (47.1)	162 (35.8)	14 (3.1)	63 (13.9)
	Hartmann procedure (*n* = 3168)	1381 (43.6)	1321 (41.7)	85 (2.7)	381 (12.0)
	Subtotal colectomy (*n* = 646)	209 (32.4)	291 (45.0)	21 (3.3)	125 (19.3)
No. of co-morbidities (includes malignancy)	0	1360 (47.2)	842 (33.5)	89 (44.3)	307 (37.3)
	1	754 (26.2)	805 (32.1)	83 (41.3)	235 (28.6)
	2	511 (17.7)	544 (21.7)	27 (13.4)	185 (22.5)
	≥ 3	255 (8.9)	319 (12.7)	2 (1.0)	95 (11.6)
ASA grade^*^	I (no systemic disease)	460 (16.0)	132 (5.3)	20 (10.0)	37 (4.5)
	II (mild systemic disease)	1168 (40.6)	564 (22.5)	62 (30.8)	180 (21.9)
	III (severe systemic disease)	917 (31.8)	865 (34.5)	79 (39.3)	312 (38.0)
	IV (severe, life-threatening)	317 (11.0)	853 (34.0)	36 (17.9)	265 (32.2)
	V (moribund)	18 (0.6)	96 (3.8)	4 (2.0)	28 (3.4)
Preoperative POSSUM score^*^	< 10	1684 (58.5)	660 (26.3)	88 (43.8)	206 (25.1)
	10–24.9	658 (22.8)	601 (23.9)	53 (26.4)	214 (26.0)
	25–49.9	315 (10.9)	570 (22.7)	32 (15.9)	189 (23.0)
	≥ 50	223 (7.7)	679 (27.1)	28 (13.9)	213 (25.9)
Urgency (h)^*^	> 18	297 (10.3)	156 (6.2)	14 (7.0)	49 (6.0)
	6–18	850 (29.5)	482 (19.2)	54 (26.9)	147 (17.9)
	< 6	1730 (60.1)	1870 (74.5)	133 (66.2)	624 (75.9)
	Missing	3 (0.1)	2 (0.1)	0 (0)	2 (0.2)
Indication for surgery^*^	Perforation only	1182 (41.0)	904 (36.0)	79 (39.3)	289 (35.2)
	Peritonitis only	698 (24.2)	512 (20.4)	42 (20.9)	180 (21.9)
	Perforation and peritonitis	1000 (34.7)	1094 (43.6)	80 (39.8)	353 (42.9)
Actual peritoneal soiling^*^	None	298 (10.3)	159 (6.3)	17 (8.5)	40 (4.9)
	Serous fluid	351 (12.2)	208 (8.3)	24 (11.9)	62 (7.5)
	Localized pus	928 (32.2)	538 (21.4)	50 (24.9)	145 (17.6)
	Free pus, blood or bowel contents	1291 (44.8)	1601 (63.8)	109 (54.2)	571 (69.5)
	Missing	12 (0.4)	4 (0.2)	1 (0.5)	4 (0.5)
Total blood loss (ml)^*^	≤500	2672 of 2849 (93.8)	2225 of 2494 (89.2)	182 of 199 (91.5)	718 of 817 (87.9)
	> 500	177 of 2849 (6.2)	269 of 2494 (10.8)	17 of 199 (8.5)	99 of 817 (12.1)
Postoperative destination^*^	Ward	1043 (36.2)	299 (11.9)	33 (16.4)	75 (9.1)
	HDU	1139 (39.5)	725 (28.9)	73 (36.3)	203 (24.7)
	ICU	695 (24.1)	1477 (58.8)	95 (47.3)	543 (66.1)
In-hospital death	Yes	172 (6.0)	685 (27.3)	22 (10.9)	150 (18.2)
	No	2708 (94.0)	1825 (72.7)	179 (89.1)	672 (81.8)

Values in parentheses are percentages of total for group unless indicated otherwise.

* At initial operation. HDU, high-dependency unit.

Surgical and medical complications following emergency laparotomy for large bowel perforation are shown in *Table 3*. The most common surgical complication was an abdominal wall/soft tissue procedure (334 patients, 5.2 per cent), which refers to the reopening, drainage or debridement of the surgical site as outlined in the surgical complication category of *Table S1*. The most common medical complications were respiratory (1574 patients, 24.5 per cent) and renal failure (1197, 18.7 per cent).

**Table 3:** Frequency of complications and in-hospital mortality by complication

	No. of patients	% of all patients	No. of in-hospital deaths	% of patients with complications who died (FTR)
**Overall**	6413		1029	16.0 (15.2, 17.0)
**Surgical complications**				
Overall	1023	16.0	172	16.8 (14.6, 19.2)
SBR	29	0.5	9	31 (15, 51)
Colectomy	62	1.0	23	37 (25, 50)
Abdominal wall/soft tissue procedure^*^	334	5.2	21	6.3 (3.9, 9.5)
Drainage (open/IR)	169	2.6	15	8.9 (5.1, 14.2)
Relook	127	2.0	36	28.3 (20.7, 37.0)
Stoma formation	71	1.1	11	15 (8, 26)
Stoma revision	88	1.4	15	17 (10, 27)
Washout	47	0.7	18	38 (25, 54)
Other procedure	96	1.5	24	25 (17, 35)
**Medical complications** ^†^				
Overall	3332	52.0	835	25.1 (23.6, 26.6)
Renal failure	1197	18.7	450	37.6 (34.8, 40.4)
Respiratory	1574	24.5	410	26.0 (23.9, 28.3)
Cardiac	434	6.8	186	42.9 (38.1, 47.7)
Stroke	47	0.7	24	51 (36, 66)
Thromboembolic	117	1.8	24	20.5 (13.6, 29.0)
Wound infection	1011	15.8	97	9.6 (7.8, 11.6)
Sepsis	940	14.7	371	39.5 (36.3, 42.7)

Values in parentheses are 95 per cent confidence intervals.

* Refers to reopening, drainage or debridement of the surgical site as outlined in the surgical complication category of *Table S1*.

† Patients can have more than one medical complication within their admission; 822 patients had both medical and surgical complications. FTR, failure to rescue; SBR, small bowel resection; IR, interventional radiology.

The overall FTR rate was 24.3 per cent (857 deaths in 3533 patients), considerably higher than the mortality rate in the 2880 patients without a complication (172 deaths, 6.0 per cent).

The risk of in-hospital death was greater among those who suffered either type of complication: 172 deaths following a surgical complication (FTR-S rate 16.8 per cent) and 835 deaths from a medical complication (FTR-M rate 25.1 per cent). Although stroke, cardiac and thromboembolic complications were relatively uncommon, the FTR rates were significant (*Table 3*).

Consequently, patients with a medical (odds ratio (OR) 5.9, 95 per cent c.i. 4.9 to 7.1) or surgical (OR 1.9, 1.2 to 3.1) complication had a significantly greater risk of death than those without a complication. Adjusting for patient factors, in-hospital mortality was significant for medical complications (OR 2.5, 2.0 to 3.0).

**Fig. 2:**
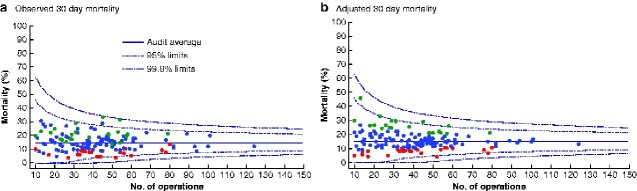
Funnel plots of observed and adjusted in-hospital mortality in 188 hospitals that performed 10 or more operations for large bowel perforation **a** Observed and **b** adjusted 30-day morality rate.

In hospitals performing 10 or more laparotomies for large bowel perforation, the adjusted in-hospital mortality rate did not demonstrate greater intrahospital variation than would be expected by chance alone (*Fig. 2*). Finally, following adjustment for patient characteristics and hospital factors, hospitals grouped as having low, medium or high overall postoperative mortality did not have different FTR rates (*P* = 0.770).

## Discussion

This study of the NELA database has shown that in-hospital mortality and overall complication rates are predictably high following emergency laparotomy for large bowel perforation. Although surgical complications were common and the FTR-S was significant, medical complications occurred considerably more frequently (52.0 per cent) and were associated with a higher risk of death (FTR-M 25.1 per cent). There was no evidence to suggest that FTR rates for these major bowel procedures varied across NHS hospitals in England.

There has been a focus on patient safety and preventable errors, particularly with the emergence of public reporting of outcomes and pay-for-performance models^22^. There are proposals in certain healthcare systems to link hospital and physician reimbursements to performance measures such as complication rates and readmissions. However, postoperative complications and mortality alone are poor indicators of performance quality as they are associated more strongly with patient factors than with hospital characteristics and delivery of care^23^^,^^24^. Moreover, complication rates often correlate poorly with in-hospital mortality owing to these differences in patient populations^24^^,^^25^. It is therefore necessary for healthcare providers to monitor reliable quality metrics that accurately reflect the care delivered^22^.

The FTR overcomes some of these limitations because it is associated less with patient characteristics and more with processes^23^^,^^25^, enabling interprovider comparisons and surgical quality assessment metrics^18^, and consequently it is better at differentiating high- and low-mortality surgical providers than postoperative complication rates^26^^,^^27^.

FTR is also better linked to system indicators associated with improved outcomes, such as hospital size, occupancy, teaching status, hospital technology, nurse-to-patient ratio, presence of more than 20 ICU beds^28–33^, physician coverage, rapid response teams, attitudes of clinical staff to culture of safety and specific behaviours^34^, enabling interprovider comparisons and surgical quality assessment metrics. The FTR is still linked to patient characteristics, such as age, presence of malignancy, presence of specific complications and frailty^35–37^.

FTR has been used in understanding hospital-level variation in in-hospital mortality following elective procedures, such as coronary artery bypass grafting^25^, abdominal aortic aneurysm repair^17^, colorectal resection^18^, pancreatectomy^38^ and oesophagectomy^39^. A recent study^40^ from the USA evaluating emergency small and large bowel resections for any pathology showed significantly higher FTR rates at higher-mortality hospitals, allowing the authors to suggest that quality improvement programmes were needed to address variations in FTR and improve outcomes^40^. In contrast, the present study failed to identify an association between FTR and hospitals. The present study focused on the common clinical entity (large bowel perforation) to reduce the heterogeneity of data and facilitate more accurate analysis. The patient cohort in this study, as well as the introduction of NELA in 2012 in England and Wales, may explain the differences between these studies. The present study also included a comprehensive list of medical and surgical complications (*Table S1*), providing a broader overview of complications that occur within emergency surgery compared with the notable previously published elective surgery FTR studies^18^^,^^25^^,^^26^^,^^39^. This addressed one of the major limitations of databases analysed previously—the under-reporting of complications—and is likely to reduce potential bias from different coding practices. Most importantly, the data are contemporaneous and show current practice nationally. The high-risk nature of the pathology (large bowel perforation) and the emergency surgery setting may also explain, in part, the reported differences in this study compared with previously published FTR studies. Another recent study^41^ showed that FTR did not identify any NHS hospitals in England with a significantly higher mortality rate following major complications after abdominal aortic aneurysm surgery.

It would seem intuitive that FTR may be more of an issue in smaller hospitals, with fewer in-house physicians and resources. The value of the NELA database is that it is representative of the population in England and Wales, because all hospitals performing emergency laparotomy are included. It also captures data on all patients undergoing emergency laparotomy for large bowel perforation, which is a limitation of other databases^27^. This prevents certain institutions, such as large teaching hospitals, being overrepresented, and reduces the under-reporting of clinical outcomes. It is also an ongoing project that provides the data to enable clinical teams to assess and benchmark the care provided against national standards, and also actively encourages teams to use the hospital-specific data to drive local quality improvement projects^42^. Clinical mechanisms underlying mortality after complications have yet to be elucidated. The ability to rescue a patient effectively from a complication relies on two distinct points of intervention: the timely recognition of a complication and the effective management of that complication.

The present study has highlighted that large bowel perforation results in numerous significant complications and FTR is high, irrespective of patient and hospital factors. Predictably, mortality following complications is also high, but does not appear to be linked to differences in FTR between hospitals. This would support the need for strategies to reduce the incidence of complications. In this study, one in six patients developed a surgical complication, whereas more than half developed a medical complication during their emergency laparotomy admission for large bowel perforation. Among ongoing efforts to reduce complication risks, in the USA, the Surgical Care Improvement Project is focusing on hospital compliance with several evidence-based processes of care. The US Centres for Medicare and Medicaid Services has enacted a policy to deny reimbursements to hospitals for so-called never events, including urinary tract infections, pressure ulcers, retained foreign bodies after surgery, and many other events^43^. The Getting it Right First Time (GIRFT) programme in the UK aims for better understanding of variations in healthcare delivery and to implement changes to promote best practice^44^. Since the inception of NELA, there has been an emphasis on outcomes following emergency laparotomy, which has resulted in a cultural shift within NHS hospitals, possibly reducing the FTR variation. This has been further reflected by the reduction in the 30-day mortality rate for all patients undergoing emergency bowel surgery, from 11.8 per cent in 2013 to 9.5 per cent in 2017, with noted improved consultant anaesthetist and surgeon presence in theatre, from 75 to 90 per cent during the same time interval^42^. In parallel, other initiatives have focused on specific processes of care, such as, intraoperative checklists^45^^,^^46^ or timely implementation of the evidence-based guidelines for the Surviving Sepsis Campaign^47^^,^^48^.

In particular, the NELA collaborative has enabled hospitals to assure the quality of their service by comparing care against the published standards and benchmarking against other hospitals, including timeliness of care, risk assessment, consultant-led delivery and a multidisciplinary approach to these high-risk patients^42^. These data are available in real-time hospitals to facilitate quality improvement. In 2018, the ‘emergency laparotomy best practice tariff’ was introduced in England and Wales, to reward hospitals that deliver care considered to be best practice, requiring assessment of risk, senior clinician involvement and critical care admission after surgery for high-risk patients^42^. Despite improvements in certain aspects, there remains a need to focus on areas, particularly in the perioperative period. For example, only one-quarter of patients with suspected sepsis requiring an emergency laparotomy currently receive antibiotics within the recommended 60 min. In addition, only 23 per cent of patients over the age of 70 years are reviewed by Healthcare of the Elderly physicians^42^. Abdominal wall/soft tissue procedures involved the most common surgical complications, and the ongoing prospective wound trials of SUNRRISE^49^ and ROSSINI 2^50^ may help to improve outcomes in this area. Furthermore, the FLO-ELA trial^51^ may help in reducing medical complications such as renal failure in the postoperative period through cardiac output monitoring to guide intravenous fluid management. Finally, the PRISM trial^52^ results are awaited to evaluate whether continuous positive airway pressure immediately after surgery can reduce respiratory complications.

Not all patient deaths following a postoperative complication will be preventable, particularly in emergency surgery^53^^,^^54^. One important metric to consider from this study is the number of deaths with no recorded complications. It is possible that measuring the rate of deaths with no recorded complications is as representative of underlying provider structures and processes as is FTR^17^, because complete avoidance of complications is also as reliant on a well functioning infrastructure as are rescuing complications when they occur^17^. These deaths with no recorded complications might represent the true number of unavoidable deaths, and FTR might be a better measure of preventable deaths^17^. Alternatively, this group may actually represent deaths after complications, but where the complication has either not been recorded clearly or has not been coded^17^.

There are limitations to this study that must be recognized. First, all causes of large bowel perforation were included. Although this produces a heterogeneous group, it is more representative of actual clinical practice for the management of this condition. Second, this was a retrospective observational study, and the NELA database includes data only for patients who required an operative intervention for large bowel perforation, and not those treated conservatively (such as patients with localized perforation), with antibiotics or who required another form of intervention. The proportion of patients turned down for surgery because of medical fitness or patient choice is also unknown. One major advantage of the NELA database is that it collects a comprehensive list of patient variables, enabling risk-adjusted outcomes and comparison to reduce selection bias^55^. In addition, restricting the study to laparotomy for large bowel perforation, rather than all forms of emergency bowel surgery, reduced the heterogeneity. Furthermore, there is some evidence of systematic under-reporting of complications utilizing HES data; however, despite this, the majority of FTR rates derived from HES were not significantly different in validation studies^17^.

Inpatient mortality and FTR rates are high following emergency laparotomy for large bowel perforation, but there is no association between FTR and hospital structures. Quality improvement efforts should focus on identifying patients at high risk of developing a complication and on the prevention of complications after emergency laparotomy.

## Collaborators

Members of the NELA Project Team are: P. Cripps, N. Eugene, J. Goodwin, S. Hare, M. Grocott, E. Davies, M. Bassett, C. Johnston, J. Lourtie, R. Moonesinghe, D. Murray, D. Papadimitriou, C. Peden, T. Poulton, T. Salih, K. Walker, S. Warren, M. Cripps, S. Drake and M. Oliver.

## Supplementary Material

zraa060_Supplementary_Data

## Notes

Presented to the International Surgical Congress of the Association of Surgeons of Great Britain and Ireland, Telford, UK, May 2019; published in abstract form as BJS 2019;**106**(Suppl 5): 64.

Members of the NELA Project Team are co-authors of this study and are listed under the heading Collaborators
