# Supplementary material for: Failure to rescue patients after emergency laparotomy for large bowel perforation: analysis of the National Emergency Laparotomy Audit (NELA)
Source: BJS Open. 2021 Feb 20;5(1):zraa060. doi: 10.1093/bjsopen/zraa060 (PMC7896807; doi:10.1093/bjsopen/zraa060)
Supplement: zraa060_Supplementary_Data [file zraa060_supplementary_data.docx]

Supplementary Table 1: OPCS-4 and ICD-10 codes used to define types of complication

| Surgical complication category | OPCS-4 codes |
| --- | --- |
| Reopening, drainage or debridement of surgical site | T301 Reopening of abdomen and re-exploration of intra-abdominal operation site and surgical arrest of postoperative bleeding  T302 Reopening of abdomen and re-exploration of intra-abdominal operation site NEC  T303 Reopening of abdomen NEC  T304 Opening of abdomen and exploration of groin  T308 Other specified opening of abdomen  T309 Unspecified opening of abdomen  T032 Reopening of chest and re-exploration of intrathoracic operation site and surgical arrest of postoperative bleeding  T033 Reopening of chest and re-exploration of intrathoracic operation site NEC  T034 Reopening of chest NEC  T414 Open removal of foreign body from peritoneum  T418 Other specified other open operations on peritoneum  T419 Unspecified other open operations on peritoneum  T424 Endoscopic removal of foreign body from peritoneum  T428 Other specified therapeutic endoscopic operations on peritoneum  T431 Diagnostic endoscopic examination of peritoneum and biopsy of lesion of peritoneum  T432 Diagnostic endoscopic examination of peritoneum and biopsy of lesion of intra-abdominal organ NEC  T438 Other specified diagnostic endoscopic examination of peritoneum  T439 Unspecified diagnostic endoscopic examination of peritoneum  T365 Creation of omental flap  T488 Other specified other operations on peritoneum  T317 Exploration of groin NEC  S608 Other specified other operations on skin  S609 Unspecified other operations on skin  S242 Local myocutaneous subcutaneous pedicle flap NEC  S604 Refashioning of scar NEC  S248 Other specified local flap of skin and muscle  S249 Unspecified local flap of skin and muscle  S57 Debridement/attention to wound of skin  T282 Suture of anterior abdominal wall  T283 Resuture of previous incision of anterior abdominal wall  S423 Secondary suture of skin NEC  S424 Resuture of skin NEC  S428 Other specified suture of skin of other site  S429 Unspecified suture of skin of other site  S472 Drainage of lesion of skin NEC  S474 Incision of lesion of skin NEC  S476 Incision of skin NEC  S478 Other specified opening of skin  S479 Unspecified opening of skin  S422 Delayed primary suture of skin NEC  S434 Removal of suture from skin NEC  S438 Other specified removal of repair material from skin  S352 Meshed split autograft of skin NEC  S358 Other specified split autograft of skin  S359 Unspecified split autograft of skin  T315 Drainage of anterior abdominal wall  T316 Removal of foreign body from anterior abdominal wall  T318 Other specified other operations on anterior abdominal wall  T774 Debridement of muscle NEC  T963 Debridement of soft tissue NEC  T964 Evacuation of seroma from soft tissue  S628 Other specified other operations on subcutaneous tissue  S629 Unspecified other operations on subcutaneous tissue  T052 Resuture of previous incision of chest wall |
| Vascular injury/complication | T553 Release fasciotomy of thigh  T554 Release fasciotomy of anterior compartment of lower leg  T555 Release fasciotomy of posterior compartment of lower leg  T556 Release fasciotomy of leg NEC  L233 Plastic repair of aorta using patch graft  L255 Operations on aortic body  L258 Other specified other open operations on aorta  L531 Repair of iliac artery NEC  L532 Open embolectomy of iliac artery  L538 Other specified other open operations on iliac artery  L621 Repair of femoral artery NEC  L622 Open embolectomy of femoral artery  L683 Repair of artery using prosthesis NEC  L684 Repair of artery using vein graft NEC  L688 Other specified repair of other artery  L689 Unspecified repair of other artery  L798 Other specified other operations on vena cava  L161 Emergency bypass of aorta by anastomosis of axillary artery to femoral artery  L208 Other specified other emergency bypass of segment of aorta  L209 Unspecified other emergency bypass of segment of aorta  L222 Revision of prosthesis of bifurcation of aorta  L223 Revision of prosthesis of abdominal aorta NEC  L224 Removal of prosthesis from aorta  L228 Other specified attention to prosthesis of aorta  L229 Unspecified attention to prosthesis of aorta  L238 Other specified plastic repair of aorta  L239 Unspecified plastic repair of aorta  L253 Open embolectomy of bifurcation of aorta  L261 Percutaneous transluminal balloon angioplasty of aorta  L262 Percutaneous transluminal angioplasty of aorta NEC  L264 Aortography  L268 Other specified transluminal operations on aorta  L269 Unspecified transluminal operations on aorta  L263 Percutaneous transluminal embolectomy of bifurcation of aorta  L634 Arteriography of femoral artery  L631 Percutaneous transluminal angioplasty of femoral artery  L632 Percutaneous transluminal embolectomy of femoral artery  L541 Percutaneous transluminal angioplasty of iliac artery  L542 Percutaneous transluminal embolectomy of iliac artery  L543 Arteriography of iliac artery  L544 Percutaneous transluminal insertion of stent into iliac artery  L58 Emergency bypass of femoral artery  L65 Revision of reconstruction of artery  L665 Percutaneous transluminal balloon angioplasty of artery  L667 Percutaneous transluminal placement of peripheral stent in artery  L701 Open embolectomy of artery NEC  L702 Open embolisation of artery NEC  L703 Ligation of artery NEC  L711 Percutaneous transluminal angioplasty of artery  L712 Percutaneous transluminal embolectomy of artery  L713 Percutaneous transluminal embolization of artery  L716 Percutaneous transluminal thrombolysis of artery  L721 Arteriography NEC  L761 Endovascular placement of one metallic stent  L762 Endovascular placement of one plastic stent  L765 Endovascular placement of three or more metallic stents  L768 Other specified endovascular placement of stent  L50 Emergency bypass of iliac artery/artery of leg  L56 Emergency replacement of aneurysmal femoral artery  L198 Other specified other replacement of aneurysmal segment of aorta  L199 Unspecified other replacement of aneurysmal segment of aorta  L974 Operations on artery NEC  L975 Operations on vein NEC  X093 Amputation of leg above knee  X094 Amputation of leg through knee  X095 Amputation of leg below knee  X098 Other specified amputation of leg  X099 Unspecified amputation of leg  X10 Amputation of foot/ankle/metatarsal bones  X11 Amputation of toe |
| Intestinal resection/stoma formation | G633 Closure of perforation of jejunum  G584 Partial jejunectomy and anastomosis of jejunum to ileum  G585 Partial jejunectomy and anastomosis of duodenum to colon  G588 Other specified excision of jejunum  G589 Unspecified excision of jejunum  G611 Bypass of jejunum by anastomosis of jejunum to jejunum  G612 Bypass of jejunum by anastomosis of jejunum to ileum  G613 Bypass of jejunum by anastomosis of jejunum to colon  G618 Other specified bypass of jejunum  G619 Unspecified bypass of jejunum  G691 Ileectomy and anastomosis of stomach to ileum  G692 Ileectomy and anastomosis of duodenum to ileum  G693 Ileectomy and anastomosis of ileum to ileum  G694 Ileectomy and anastomosis of ileum to colon  G698 Other specified excision of ileum  G699 Unspecified excision of ileum  G711 Bypass of ileum by anastomosis of jejunum to ileum  G712 Bypass of ileum by anastomosis of ileum to ileum  G713 Bypass of ileum by anastomosis of ileum to caecum  G714 Bypass of ileum by anastomosis of ileum to transverse colon  G715 Bypass of ileum by anastomosis of ileum to colon NEC  G718 Other specified bypass of ileum  G719 Unspecified bypass of ileum  G721 Anastomosis of ileum to caecum  G724 Anastomosis of ileum to rectum  G731 Revision of anastomosis of ileum  G734 Resection of ileocolic anastomosis  G738 Other specified attention to connection of ileum  G739 Unspecified attention to connection of ileum  G762 Open relief of strangulation of ileum  G763 Open relief of obstruction of ileum NEC  G768 Other specified intra-abdominal manipulation of ileum  G769 Unspecified intra-abdominal manipulation of ileum  G782 Strictureplasty of ileum  G784 Closure of perforation of ileum  G786 Open intubation of ileum  G788 Other specified other open operations on ileum  G824 Intubation of ileum NEC  G822 Intubation of ileum for decompression of intestine  G828 Other specified other operations on ileum  G511 Bypass of duodenum by anastomosis of stomach to jejunum  G512 Bypass of duodenum by anastomosis of duodenum to duodenum  G513 Bypass of duodenum by anastomosis of duodenum to jejunum  G632 Incision of jejunum  G638 Other specified other open operations on jejunum  G639 Unspecified other open operations on jejunum  T374 Repair of mesentery of small intestine  G671 Intubation of jejunum for decompression of intestine  G674 Intubation of jejunum NEC  G728 Other specified other connection of ileum  G741–G749 Creation of ileostomy  G601 Creation of jejunostomy  G618 Other specified bypass of jejunum  G619 Unspecified bypass of jejunum  G729 Unspecified other connection of ileum  G789 Unspecified other open operations on ileum  G608 Other specified artificial opening into jejunum  G609 Unspecified artificial opening into jejunum  H168 Other specified incision of colon  H162 Caecotomy  T384 Repair of mesentery of colon  T388 Other specified operations on mesentery of colon  T398 Other specified operations on posterior peritoneum  P253 Repair of rectovaginal fistula  H151 Loop colostomy  H152 End colostomy  H158 Other specified other exteriorisation of colon  H159 Unspecified other exteriorisation of colon  H141 Tube caecostomy  H148 Other specified exteriorisation of caecum  H149 Unspecified exteriorisation of caecum  H161 Drainage of colon  H163 Colotomy  H041 Panproctocolectomy and ileostomy  H052 Total colectomy and ileostomy and creation of rectal fistula HFQ  H053 Total colectomy and ileostomy NEC  H058 Other specified total excision of colon  H059 Unspecified total excision of colon  H06 Extended right hemicolectomy  H07 Right hemicolectomy  H08 Transverse colectomy  H09 Left hemicolectomy  H10 Sigmoid colectomy  H11 Colectomy  H298 Other specified subtotal excision of colon  H299 Unspecified subtotal excision of colon  H305 Irrigation of colon  H308 Other specified other operations on colon  H331 Abdominoperineal excision of rectum and end colostomy  H332 Proctectomy and anastomosis of colon to anus  H333 Anterior resection of rectum and anastomosis of colon to rectum using staples  H334 Anterior resection of rectum and anastomosis NEC  H335 Rectosigmoidectomy and closure of rectal stump and exteriorisation of bowel  H336 Anterior resection of rectum and exteriorization of bowel  H338 Other specified excision of rectum  H339 Unspecified excision of rectum  H13 Colonic bypass  H193 Enterorrhaphy of colon  H198 Other specified other open operations on colon  H199 Unspecified other open operations on colon  H175 Open relief of strangulation of colon  H176 Open relief of obstruction of colon NEC  H178 Other specified intra-abdominal manipulation of colon  H179 Unspecified intra-abdominal manipulation of colon  H192 Fixation of colon  H624 Intubation of bowel NEC  H628 Other specified other operations on bowel  H629 Unspecified other operations on bowel  H28 Endoscopic examination of sigmoid colon |
| Medical complication category | ICD-10 codes |
| Renal complication | N17 Acute renal failure  N19 Unspecified kidney failure  N990 Postprocedural renal failure |
| Respiratory complication | J960 Acute respiratory failure  J969 Respiratory failure, unspecified  J13 Pneumonia due to *Streptococcus pneumoniae*  J14 Pneumonia due to *Haemophilus influenzae*  J15 Bacterial pneumonia NEC  J16 Pneumonia due to other infectious organisms NEC  J18 Pneumonia organism unspecified  J22 Unspecified acute lower respiratory infection  J69 Pneumonitis due to solids and liquids  J981 Pulmonary collapse  J80 Adult respiratory distress syndrome  J182 Hypostatic pneumonia unspecified |
| Cardiac complication | I21 Acute myocardial infarction  I501 Left ventricular failure  I500 Congestive heart failure  I509 Heart failure unspecified  J81 Pulmonary oedema  I248 Other forms of acute ischaemic heart disease  R570 Cardiogenic shock |
| Neurological complication/stroke | I63 Cerebral infarction |
| Thromboembolism | I801 Phlebitis and thrombophlebitis of femoral vein  I802 Phlebitis and thrombophlebitis of other deep vessels of lower extremities  I803 Phlebitis and thrombophlebitis of lower extremities unspecified  I26 Pulmonary embolism |
| Wound infection | T814 Infection following a procedure NEC  T813 Disruption of operation wound NEC  T815 Foreign body accidentally left in body cavity or operation wound following a procedure |

NEC, not elsewhere classified; HFQ, however further quantified.
